# A new crane fly species of the genus *Libnotes* Westwood, 1876 (Diptera, Limoniidae) from Jilin, China

**DOI:** 10.3897/BDJ.10.e87316

**Published:** 2022-10-17

**Authors:** Yuanyuan Xu, Yilin Yao, Peifu Zhang, Runze Zheng, Xiao Zhang

**Affiliations:** 1 Guangxi key laboratory of Agric-Environment and Agric-Products Safety and National Demonstration Center for Experimental Plant Science Education, Agricultural College, Guangxi University, Nanning 530004, China Guangxi key laboratory of Agric-Environment and Agric-Products Safety and National Demonstration Center for Experimental Plant Science Education, Agricultural College, Guangxi University Nanning 530004 China; 2 Shandong Engineering Research Center for Environment-Friendly Agricultural Pest Management, College of Plant Health and Medicine, Qingdao Agricultural University, Qingdao 266109, China Shandong Engineering Research Center for Environment-Friendly Agricultural Pest Management, College of Plant Health and Medicine, Qingdao Agricultural University Qingdao 266109 China; 3 Fangzi Bureau of Agriculture and Rural Affairs, Weifang 261200, China Fangzi Bureau of Agriculture and Rural Affairs Weifang 261200 China

**Keywords:** Chinese fauna, new species, new record, taxonomy, Limoniinae

## Abstract

**Background:**

Twenty-eight *Libnotes* Westwood, 1876 species belonging to three subgenera have been known to occur in China, of which 13 belong to the nominotypical subgenus. Amongst the 13 Chinese *Libnotes* (s. str.) species, eight are from the Chinese mainland and five are from Taiwan.

**New information:**

A *Libnotes* (s. str.) species from Jilin, China, L. (L.) changbaishana **sp. nov.** is described and illustrated as new to science. The genus *Libnotes* is recorded from Jilin Province for the first time. The new species can be distinguished from congeners mainly by its body colour, wing and male genitalia.

## Introduction

*Libnotes* Westwood, 1876 is a species-rich limoniid genus with a total number of 295 species/subspecies, known from the Oriental (131 species/subspecies), Australasian/Oceanian (110 species/subspecies), Afrotropic (40 species/subspecies) and Palaearctic (26 species) Regions (Oosterbroek 2022). Members of the genus are grouped into eight subgenera: *Afrolimonia* Alexander, 1965, *Goniodineura* van der Wulp, 1895, *Gressittomyia* Alexander, 1936, *Laosa* Ed­wards, 1926, *Libnotes* (s. str.), *Metalibnotes* Alexander, 1972, *Neolibnotes* Alexander, 1972 and *Paralibnotes* Alexander, 1972. Most *Libnotes* species are xylophilous at the larval stage and may have different preferences for wood decaying conditions (Krivosheina 2008, Podenas et al. 2015), but it is also reported that L. (L.) puella Alexander, 1925 can be herbivorous (Suetsugu et al. 2019).

In the past decade, a large number of taxonomic studies have been carried out on the genus *Libnotes* in Asia, mainly focusing on the species of China (Men 2015, Kang and Zhang 2021, Zhang and Yang 2022), Korea (Podenas et al. 2015, Podenas 2016, Podenas and Byun 2018) and Japan (Kato et al. 2016). Twenty-eight *Libnotes* crane flies belonging to three subgenera have been recorded from China, of which 13 belong to the nominotypical subgenus, eight belong to the subgenus Goniodineura, six belong to the subgenus Laosa and one belongs to the subgenus Gressittomyia (Oosterbroek 2022). Amongst the 13 Chinese *Libnotes* (s. str.) species, eight are from the Chinese mainland and five are from Taiwan, but this is unlikely to be the final number. In this study, a new *Libnotes* (s. str.) species from Jilin Province is added to the fauna of Chinese mainland, which also represents a new record genus of Jilin, China. Description and illustration of the new species are presented.

## Materials and methods

Specimens for this study were collected in Jilin, China in 2014 and deposited in the Entomological Museum of China Agricultural University, Beijing, China (CAU) and the Entomological Museum of Qingdao Agricultural University, Shandong, China (QAU). The holotype and paratype of L. (L.) basistrigata (Alexander, 1934), deposited in the National Museum of Natural History, Smithsonian Institution, Washington, DC, USA (USNM), were also examined. Genitalic preparations of male was made by macerating the apical portion of the abdomen in cold 10% sodium hydroxide (NaOH) for 12–15 hours. Observations and illustrations were made using a ZEISS Stemi 2000–C stereomicroscope. Photographs were taken with a Canon EOS 90D digital camera through a macro lens. Details of colouration were examined in specimens immersed in 75% ethanol (C_2_H_5_OH).

The morphological terminology mainly follows Cumming and Wood (2017) and that for ve­nation follows de Jong (2017). The following abbreviations in figures are used: aed = aedeagus, cerc = cercus, goncx = gonocoxite, hyp vlv = hypogynial valve, i gonst = inner gonostylus, o gonst = outer gonostylus, pm = paramere, rp = rostral prolongation, st 8 = sternite 8, tg 9 = tergite 9, tg 10 = tergite 10.

## Taxon treatments

### Libnotes (Libnotes) changbaishana

Xu and Zhang sp. n.

04F330F2-CC78-5BAD-B7CD-AE09B9E29AD7

1E60C684-E3E8-4D73-823A-CDA8C4A3FCFE

#### Materials

- **Type status:** Holotype. **Occurrence:** recordedBy: Zehui Kang (collector); individualCount: 1; sex: male; lifeStage: adult; occurrenceID: 70F5CFFF-C990-5D3B-90BC-15CD65795F92; **Taxon:** class: Insect; order: Diptera; family: Limoniidae; genus: Libnotes; subgenus: Libnotes; **Location:** country: China; stateProvince: Jilin; county: Antu; locality: Mount Changbaishan, Erdaobaihe; **Event:** year: 2014; month: September; day: 11; **Record Level:** institutionCode: CAU; basisOfRecord: PreservedSpecimen
- **Type status:** Paratype. **Occurrence:** recordedBy: Zehui Kang (collector); individualCount: 1; sex: female; lifeStage: adult; occurrenceID: 9C58B9F7-6BC8-5F26-8EDD-046DFA95DC90; **Taxon:** class: Insect; order: Diptera; family: Limoniidae; genus: Libnotes; subgenus: Libnotes; **Location:** country: China; stateProvince: Jilin; county: Antu; locality: Mount Changbaishan, Erdaobaihe; **Event:** year: 2014; month: September; day: 11; **Record Level:** institutionCode: QAU; basisOfRecord: PreservedSpecimen

#### Description

**Diagnosis.** Antenna with scape dark brown, pedicel brown, first flagellomere brown and remaining flagellomeres brownish-yellow. Prescutum and presutural scutum uniformly dark brown. Pleuron variegated. Femora and tibiae yellow with tips brownish-black. Sc long, ending beyond fork of Rs; m-m 1.5 times as long as basal section of M_3_; m-cu about equal in length beyond fork of M and at about 1/7 of cell dm. Abdominal tergites with a dark brown longitudinal stripe at middle. Inner gonostylus slightly longer than outer gonostylus.

**Figure 1.**
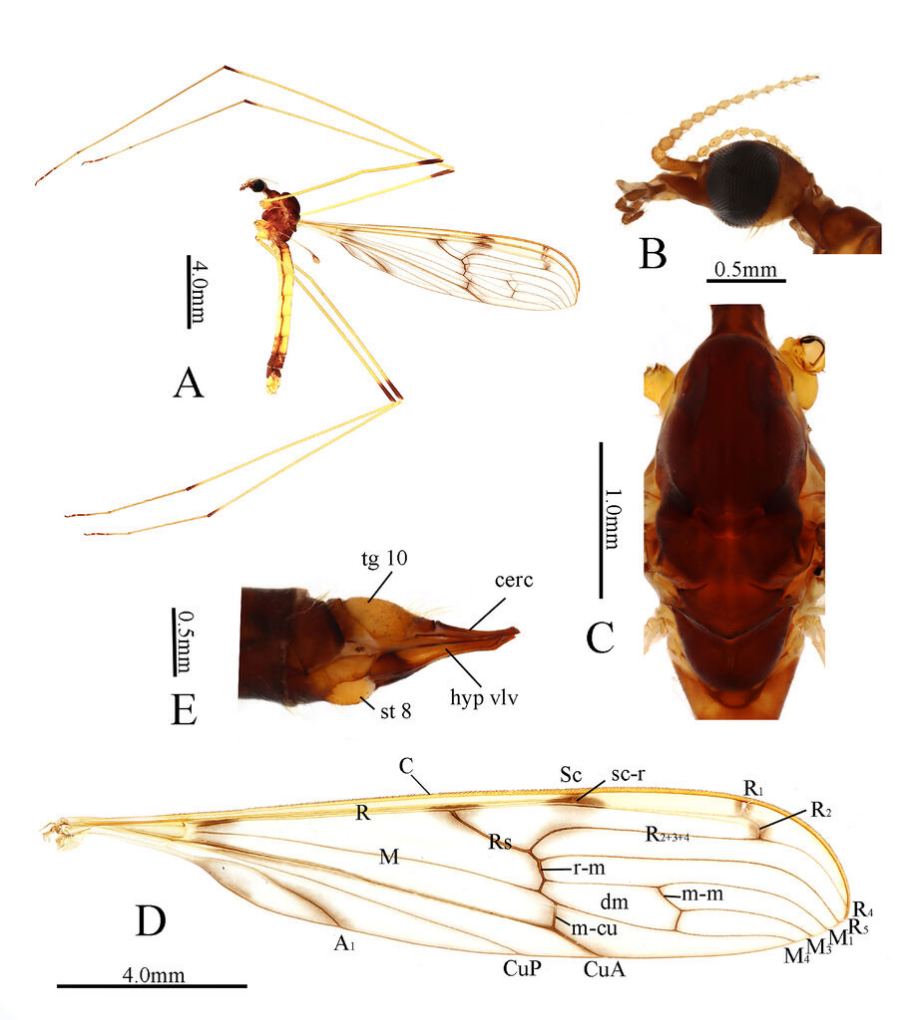
Libnotes (Libnotes) changbaishana sp. nov. **A** habitus of male, lateral view; **B** head, lateral view; **C** thorax, dorsal view; **D** wing; **E** female ovipositor, lateral view.

**Figure 2.**
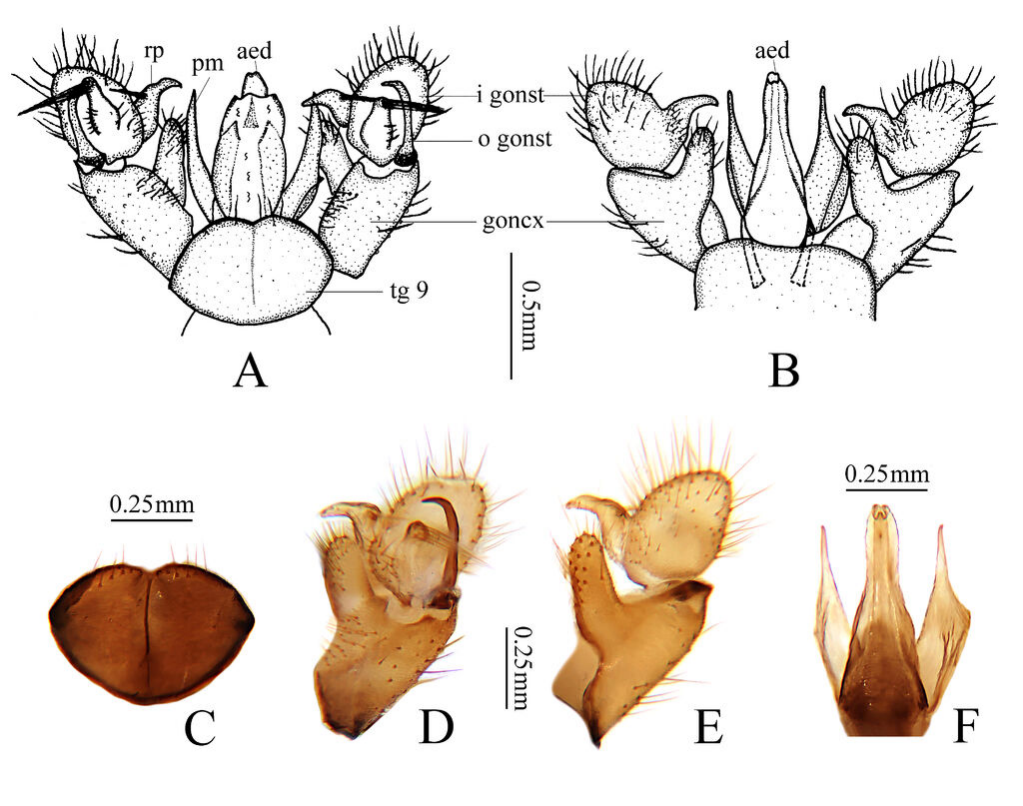
Libnotes (Libnotes) changbaishana sp. nov. **A** male hypopygium, dorsal view; **B** male hypopygium, ventral view; **C** tergite 9, dorsal view; **D** gonocoxite, outer gonostylus and inner gonostylus, dorsal view; **E** gonocoxite and inner gonostylus, ventral view; **F** aedeagus and parameres, ventral view.

**Male** (Figs. 1A–D and 2). Body length 13.0 mm, antenna length 2.4 mm, wing length 17.0 mm, halter length 3.0 mm.

Head (Fig. 1B). Dark brown. Setae on head dark brown. Antenna with scape dark brown; pedicel brown; first flagellomere brown, remaining flagellomeres brownish-yellow. Scape long cylindrical, 4 times as long as wide; pedicel oval, widened distally; flagellomeres subcylindrical, apically tapering and elongated. Rostrum brownish-yellow with basal half dark brown; palpus dark brown. Setae on rostrum and palpus dark brown.

Thorax (Fig. 1C). Pronotum dark brown. Prescutum and presutural scutum uniformly dark brown. Postsutural scutum dark brown, paler medially and laterally. Scutellum dark brown, slightly paler medially. Mediotergite dark brown. Pleuron (Fig. 1A) variegated, middles of anepisternum and katepisternum dark brown, middles of metanepisternum and metakatepisternum brown. Coxae and trochanters brownish-yellow; femora and tibiae yellow with tips brownish-black; tarsi brownish-yellow, tip of each tarsus dark brown. Setae on legs brown. Wing (Fig. 1D) tinged with brownish-yellow, except yellow costal field; small, but distinct brown spots surround R_2_ and tip of R_1_ and distinct brown patterns surround sc-r, tip of Sc, fork of Rs, base of R_2+3+4_, r-m, base of dm cell, m-m, base of M_3_, m-cu, tip of CuA, distal half of A_1_ and sub-base of wing (just posterior to crossvein h). Veins at costal field yellow, remaining veins brown, but dark brown in darkened areas and at branching points. Venation: Sc long, ending beyond fork of Rs, sc-r close to tip of Sc; Rs slightly curved; m-m 1.5 times as long as basal section of M_3_; m-cu about equal in length beyond fork of M and at about 1/7 of cell dm. Halter pale brownish-yellow with knob brown.

Abdomen. Tergite 1 dark brown; tergites 2–7 yellow, tergites 8–9 dark brown; tergites 1–8 with dark brown lateral borders and a dark brown longitudinal stripe at middle. Sternite 1 pale brown; sternites 2–6 yellow; sternite 7 brown; sternites 8–9 dark brown. Setae on abdomen white.

Hypopygium (Fig. 2). Yellow. Tergite 9 (Fig. 2A and C) nearly oval-shaped, transverse plate. Lateral margins darkened, narrowing, posterior margin provided with setae, with small me­dian emargination. Gonocoxite (Fig. 2A, B, D and E) stubby, with elongated, setose ventromesal lobe. Outer gonostylus (Fig. 2A and D) narrow, sclerotised, distally curved with acute tip. Inner gonostylus (Fig. 2A, B, D and E) slightly longer than outer gonostylus, nearly oval. Rostral prolongation arched, dilated at base, becoming narrow towards obtuse apex, basally bearing tubercle that is armed with two spines. Another nearly oval lobe starts near base of gonostylus, apically bearing six setae, that are at right angles to the lobe. Paramere elongated, wide at base, triangular distally, apex narrow. Aedeagus long, slightly narrowed at middle (Fig. 2A, B and F).

**Female**. Body length 16.0 mm, wing length 18.5 mm. Similar to male. Ovipositor with tergite 10 brownish-yellow, distal margin darker brown (Fig. 1E). Cercus brown, nearly straight, distal part slightly raised, tip acute, with dorsal pre-apical bump. Sternite 8 with basal third brown, rest of sclerite pale brownish-yellow. Hypogynial valve brownish-yellow with base dark brown.

#### Etymology

The specific name *changbaishana* (adjective, feminine) referring to the type locality, Mount Changbaishan.

#### Distribution

Known only from the type locality (Jilin, China).

#### Taxon discussion

This species is somewhat similar to L. (L.) nohirai Alexander, 1918 from Russia, North Korea, South Korea and Japan in having similar wing venation and colourations of pleuron and leg, but it can be distinguished from the latter by the antenna with brownish-yellow flagellomeres, the uniformly dark brown prescutum and presutural scutum, the dark band just posterior to crossvein h and the inner gonostylus of the male genitalia being longer than outer gonostylus. In L. (L.) nohirai, the flagellomeres of the antenna are light yellow, the prescutum and presutural scutum are light brownish-yellow with four brown longitudinal stripes, there is no dark band just posterior to crossvein h and the inner gonostylus of the male genitalia is shorter than the outer gonostylus (Alexander 1918, Savchenko 1983). This species is also somewhat similar to L. (L.) basistrigata from North Korea, South Korea and China in having similar wing pattern and colouration of leg, but it can be easily distinguished from the latter by the uniformly dark brown prescutum and presutural scutum, the variegated pleuron, the wing with the m-m 1.5 times as long as the basal section of M_3_, the inner gonostylus being longer than the outer gonostylus and sternite 8 of female being pale brownish-yellow with brown basal 1/3. In L. (L.) basistrigata, the prescutum and presutural scutum are uniformly pale yellow, the pleuron is uniformly pale yellow, the m-m of wing is twice as long as the basal section of M_3_, the inner gonostylus is shorter than outer gonostylus (Alexander 1934) and sternite 8 of the female being uniformly brownish-yellow (Podenas et al. 2015).

This species are closely related to L.(L.) longistigma Alexander, 1921 in the key to the Chinese *Libnotes* (s. str.) species in Men (2015), but it can be distinguished from the latter by the wing with the m-m as long as the basal section of M_3_ and the inner gonostylus of the male genitalia being longer than outer gonostylus. In L. (L.) longistigma, the m-m of wing is twice as long as the basal section of M_3_ and the inner gonostylus of the male genitalia is shorter than the outer gonostylus (Savchenko and Krivolutskaya 1976, Podenas et al. 2015).

## Supplementary Material

XML Treatment for Libnotes (Libnotes) changbaishana
